# *Solenopsis invicta virus 3*: Mapping of Structural Proteins, Ribosomal Frameshifting, and Similarities to *Acyrthosiphon pisum virus* and *Kelp fly virus*

**DOI:** 10.1371/journal.pone.0093497

**Published:** 2014-03-31

**Authors:** Steven M. Valles, Susanne Bell, Andrew E. Firth

**Affiliations:** 1 Center for Medical, Agricultural and Veterinary Entomology, Agricultural Research Service, United States Department of Agriculture (USDA-ARS), Gainesville, Florida, United States of America; 2 Department of Pathology, University of Cambridge, Cambridge, United Kingdom; Kantonal Hospital St. Gallen, Switzerland

## Abstract

*Solenopsis invicta virus 3* (SINV-3) is a positive-sense single-stranded RNA virus that infects the red imported fire ant, *Solenopsis invicta*. We show that the second open reading frame (ORF) of the dicistronic genome is expressed via a frameshifting mechanism and that the sequences encoding the structural proteins map to both ORF2 and the 3' end of ORF1, downstream of the sequence that encodes the RNA-dependent RNA polymerase. The genome organization and structural protein expression strategy resemble those of *Acyrthosiphon pisum virus* (APV), an aphid virus. The capsid protein that is encoded by the 3' end of ORF1 in SINV-3 and APV is predicted to have a jelly-roll fold similar to the capsid proteins of picornaviruses and caliciviruses. The capsid-extension protein that is produced by frameshifting, includes the jelly-roll fold domain encoded by ORF1 as its N-terminus, while the C-terminus encoded by the 5' half of ORF2 has no clear homology with other viral structural proteins. A third protein, encoded by the 3' half of ORF2, is associated with purified virions at sub-stoichiometric ratios. Although the structural proteins can be translated from the genomic RNA, we show that SINV-3 also produces a subgenomic RNA encoding the structural proteins. Circumstantial evidence suggests that APV may also produce such a subgenomic RNA. Both SINV-3 and APV are unclassified picorna-like viruses distantly related to members of the order Picornavirales and the family *Caliciviridae*. Within this grouping, features of the genome organization and capsid domain structure of SINV-3 and APV appear more similar to caliciviruses, perhaps suggesting the basis for a "Calicivirales" order.

## Introduction

**Figure 1:**
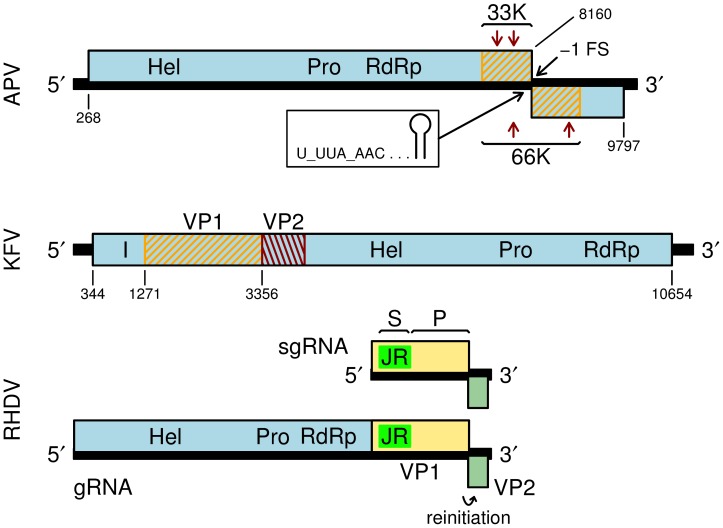
Genome maps for *Acyrthosiphon pisum virus* (APV), *Kelp fly virus* (KFV), and *Rabbit hemorrhagic disease virus* (RHDV). RHDV is a calicivirus in the genus *Lagovirus*. Helicase (Hel), protease (Pro) and RNA-dependent RNA polymerase (RdRp) domains are indicated. A predicted inhibitor of apoptosis binding domain in KFV is indicated with an I [5]. The APV structural proteins 33K and 66K (orange hatching; boundaries estimated based on protein sizes) are encoded near the 3' end of the genome; expression of 66K depends on a -1 ribosomal frameshift (-1 FS). Arrows indicate locations of 33K and 66K peptides detected via Edman degradation of cyanogen bromide cleavage products [2]. The N-termini of the KFV structural proteins VP1 and VP2 have been mapped via Edman degradation; the C-terminal extent of VP2 is estimated from its gel mobility [5]. The calicivirus structural proteins VP1 (tan) and VP2 (olive green) are expressed from a sgRNA. The N-terminal part of VP1 contains a picorna-like jelly-roll fold shell (S) domain ('JR'; bright green), while the C-terminal part comprises a protruding (P) domain.

*Acyrthosiphon pisum virus* (APV) is an unclassified picorna-like positive-sense single-stranded RNA virus that infects the pea aphid, *Acyrthosiphon pisum* [1], [2]. The approximately 10-kb genomic RNA is polyadenylated and contains two long ORFs (Fig. 1). The 5' ORF (ORF1) is predicted to encode the nonstructural proteins since its product contains canonical motifs for helicase, protease and RNA-dependent RNA polymerase (RdRp) domains. Purified virions contain proteins with estimated masses of approximately 66, 33, 24 and 23 kDa, where the most abundant protein, 33K, is estimated to occur at 5- to 10-fold excess relative to the other capsid-associated proteins [1]. The genomic regions encoding the capsid proteins have been partially mapped via Edman degradation of five internal peptides. Unusually, the 33K and 23/24K proteins map to the 3' end of ORF1 while the 66K protein maps to both the 3' end of ORF1 and the 5' half of ORF2 (Fig. 1); thus the 66K protein is a transframe protein. The presence of canonical motifs (a U_UUA_AAC slippery site and 3'-proximal predicted RNA stem-loop structure; Fig. 1) [3] for programmed -1 ribosomal frameshifting in the short region where ORF1 and ORF2 overlap, strongly suggests that the mechanism by which the 66K transframe protein is produced is ribosomal frameshifting [2].

*Kelp fly virus* (KFV), another unclassified picorna-like virus, was described in 1976 and its genome sequence was reported in 2005 [4], [5]. The virus has a positive-sense single-stranded RNA genome of ∼11 kb, that contains a single long ORF whose product contains canonical motifs for helicase, protease and RdRp domains, encoded within the 3' approximately two thirds of the ORF. Structural proteins VP1 and VP2 (see below), and a predicted inhibitor of apoptosis (IAP)-like domain, are encoded within the 5'-terminal one third of the ORF (Fig. 1). VP1 was observed to bear similarities to the APV structural proteins, in particular in the N-terminal one third (which corresponds to the APV 33K protein). Virions are 29±1 nm and have high buoyant density (1.425±0.002 g/ml) in neutral CsCl [4]. Further analysis revealed the presence of two major structural proteins with sizes ∼75 kDa and ∼29 kDa in the approximate ratio 1∶5, besides a minor VP1-related product of ∼16 kDa, and a minor product that was slightly smaller than the 75 kDa product. Edman degradation precisely mapped the N-termini of the VP1 and VP2 structural proteins, and mass spectrometric analysis mapped internal peptides. Cryoelectron microscopy and image reconstruction revealed an icosahedral structure with turrets at some of the 12 five-fold vertices, though on average only ∼25% of five-fold vertices were estimated to be occupied by turrets. Based on volumetric calculations, the capsid shell was proposed to comprise 60 copies of an asymmetric unit comprising a protein or proteins of approximately 75 kDa, while the turret domain was estimated to comprise a unit of approximately 70 kDa. It was proposed that VP1 might represent the turret domain, with a footprint in the shell, while the bulk of the shell might comprise VP2 [5].

*Solenopsis invicta virus 3* (SINV-3) infects the red imported fire ant *Solenopsis invicta* [6]. SINV-3 has a positive-sense single-stranded RNA genome of ∼10.4 kb. The genomic RNA is polyadenylated and contains two long ORFs. The product of the 5' ORF contains canonical motifs for helicase, protease and RdRp domains, but the structural proteins had not been mapped, nor had the identity of the protein or proteins encoded by ORF2 been determined. SINV-3 was observed to bear amino acid sequence similarity to KFV in the helicase, protease and RdRp domains, although the two viruses are nonetheless highly divergent. SINV-3 was also observed to bear similarity to KFV in particle morphology, with icosahedral 27.3±1.3 nm virions with apparent surface projections and high buoyant density (1.39±0.02 g/ml).

In contrast to APV, KFV and SINV-3, members of the family *Caliciviridae* infect vertebrates. Caliciviruses have positive-sense single-stranded RNA genomes typically of ∼7.5–8.5 kb, and have not yet been assigned to an order. Viral RNAs are polyadenylated and bear a 5'-covalently linked protein, VPg, acquired via its role as a protein primer for the viral polymerase (reviewed in [7]). Unlike picornaviruses, caliciviruses have short 5' UTRs without internal ribosome entry site (IRES) elements and VPg plays a role in ribosome recruitment. The non-structural proteins are encoded within a long ORF covering the 5'-proximal approximately two thirds of the genome, while the major capsid protein (VP1) and a small basic protein (VP2) are encoded within two 3'-proximal ORFs. VP2 is a minor component of the virion [8]. The nonstructural proteins are translated from the genomic RNA, while VP1 and VP2 are translated from a subgenomic RNA (sgRNA), where translation of VP2 depends on a termination-reinitiation mechanism: due to special signals within the mRNA, a proportion (estimated to be ∼5–20%) of ribosomes that have terminated after translating VP1 are then able to reinitiate on the VP2 initiation codon and translate VP2 [9]. Interestingly, in some genera (e.g. *Lagovirus* and *Sapovirus*), the VP1 ORF is contiguous with the nonstructural polyprotein ORF, so that some VP1 is also translated from the genomic RNA as a fusion with the nonstructural polyprotein, even though VP1 is also translated from the sgRNA (Fig. 1). Further, in at least one species where these ORFs are not contiguous, some VP1 is translated from the genomic RNA via termination-reinitiation [10]. Thus it has been proposed that there may be a selective advantage in producing a small amount of capsid protein early in infection, and indeed VP1 has been shown to enhance RdRp activity [10], [11]. Virions have *T* = 3 symmetry and comprise 180 copies of VP1 and perhaps 1 to 8 copies of VP2. VP2 is thought to be located inside the virion and appears to be involved in virus assembly and stability [12], [13], [14], [15], [16]. The N-terminal approximately one third of VP1 contains a picorna-like jelly-roll fold shell domain (S), while the C-terminal approximately two thirds comprises a protruding domain (P); P and S are connected by a flexible hinge. The S domains form the virion shell while the P domains form protrusions on the virion surface [17], [18], [19], [20].

The sequences encoding the SINV-3 structural proteins had not been previously mapped. Here we show that they map to ORF2 and the 3' end of ORF1. We show that expression of ORF2 depends on frameshifting, and we show that a sgRNA encoding the structural proteins is produced in SINV-3-infected ants. We also discuss similarities between SINV-3, APV, KFV and the caliciviruses.

## Results

### Sequence analysis suggests that APV expresses its structural proteins from a subgenomic RNA

**Figure 2:**
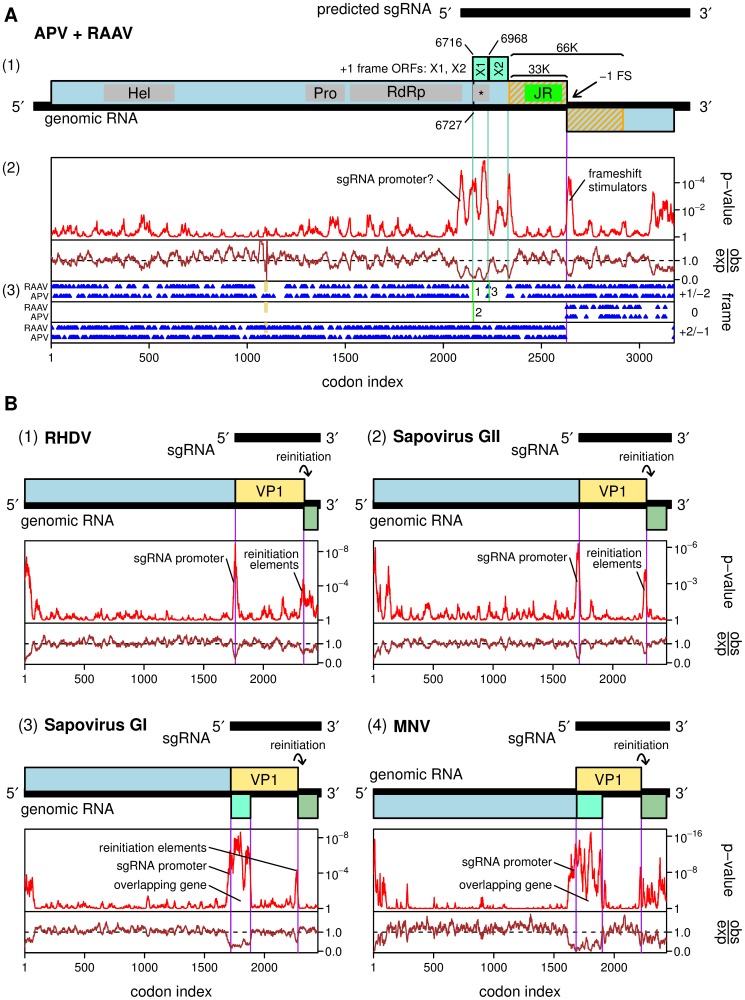
Comparative genomic analysis of *Acyrthosiphon pisum virus* (APV) and *Rosy apple aphid virus* (RAAV). **A.** (1) Map of the APV genome. The 33K structural protein contains a predicted picorna-like jelly-roll fold domain ('JR'; bright green). A predicted dsRNA binding domain is indicated with an asterisk. Potential overlapping genes X1 and X2 are indicated in turquiose. (2) Analysis of conservation at synonymous sites between APV and RAAV. The lower panel (brown) shows the ratio of the observed number of substitutions to the number expected under a null model of neutral evolution at synonymous sites, while the upper panel (red) shows the corresponding *p*-value. (3) Positions of stop codons (blue) in each of the three forward reading frames. Positions of alignment gaps are indicated in tan. The vertical green lines labeled '1', '2' and '3' indicate the positions and frames of the first three AUG codons on the predicted sgRNA. **B.** Genome maps and synonymous site conservation analyses for four calicivirus clades: (1) *Rabbit hemorrhagic disease virus* (RHDV; genus *Lagovirus*; 33 sequences), (2) group II sapoviruses (5 sequences), (3) group I sapoviruses (9 sequences), and (4) *Murine norovirus* (MNV; 58 sequences). VP1 and VP2 are indicated in tan and olive green, respectively; overlapping genes in group I sapoviruses and MNV are indicated in turquoise. All plots use a 25-codon sliding window, except MNV where a 15-codon sliding window is used. Note that *p*-values cannot be directly compared between plots as larger and more diverse alignments provide more statistical power (cf. the MNV and APV/RAAV plots).

Although the APV genome was published in 1997, it was not until 2009 that the genome of a second closely related virus, *Rosy apple aphid virus* (RAAV), was published [21]. RAAV has the same genome organization as APV and retains the U_UUA_AAC frameshift site in the ORF1-ORF2 overlap region. The two viruses share on average 87% amino acid identity. We analyzed the degree of nucleotide conservation between APV and RAAV at synonymous sites using techniques described previously (Fig. 2A) [22]. Regions of enhanced conservation at synonymous sites are indicative of overlapping functional elements - either coding or non-coding [23], [24]. The analysis revealed a number of regions of enhanced conservation (Fig. 2A). A region of conservation immediately 3'-adjacent to the frameshift site corresponds to the predicted frameshift-stimulatory RNA stem-loop structure in APV [2], that is also preserved in RAAV. A region of conservation at the 3' end of ORF2 may correspond to replicational elements as such features are common in this genomic location in many RNA viruses (e.g. [24], [25]). Interestingly, high synonymous site conservation was also observed some distance upstream of the 3' end of ORF1, covering an extended region between the RdRp- and capsid-encoding regions of ORF1 (Fig. 2A).

Previously we and others have observed similar patterns of conservation in alignments of, for example, calicivirus sequences (Fig. 2B) [26]. In those genera where the nonstructural polyprotein and VP1 ORFs are fused (e.g. *Lagovirus* and *Sapovirus*), a prominent peak in synonymous site conservation marks the junction between the sequence encoding the nonstructural polyprotein and the sequence encoding VP1 (Fig. 2B, panels 1 and 2). The enhanced conservation presumably reflects the requirement for the sequence in this region to simultaneously encode protein and contain sgRNA promoter elements [26], [27]. In some cases, translation enhancer elements on the sgRNA may also contribute to increased synonymous site conservation. Similarly, in *Murine norovirus* (genus *Norovirus*, genogroup GV), where the nonstructural polyprotein and VP1 ORFs are separate, there is enhanced synonymous site conservation at the 3' end of the nonstructural polyprotein ORF, starting from ∼200 nt upstream of the sgRNA start site (Fig. 2B, panel 4) [26]. In several calicivirus species, an additional accessory protein is encoded by a short ORF overlapping the 5'-proximal region of the VP1 ORF. In *Murine norovirus*, an ORF of ∼213 codons in this location encodes a virulence factor, VF1 [28]. The presence of this ORF leads to an extended region of synonymous site conservation in the corresponding part of the VP1 ORF (Fig. 2B, panel 4) [26]. Group I sapoviruses are also thought to harbour an overlapping ORF in this genomic location (Fig. 2B, panel 3) [26], [29]. Such accessory ORFs are thought to be translated, along with VP1, from the sgRNA via leaky scanning mechanisms (reviewed in [3]).

These observations suggest an explanation for the region of synonymous site conservation in APV and RAAV - namely that it corresponds to a promoter sequence for producing a sgRNA from which the structural proteins could be expressed, and possibly also one or more overlapping ORFs from which accessory proteins might be translated from the same sgRNA. This proposal correlates well with the observation of a sub-genome length (4-kb) RNA by Northern blot analysis using a probe to nucleotides 4959–10016 of the APV genome [2]. The 4-kb RNA was found to be more abundant than genomic RNA in APV-infected aphids but less abundant in purified virions. Van der Wilk et al. postulated that the 4-kb RNA could be either a sgRNA or a defective interfering RNA [2]. (The latter explanation seemed attractive because the 4-kb RNA also weakly hybridized to a 5'-terminal cDNA clone, and also because the capsid protein could be expressed from the genomic RNA so a sgRNA seemed superfluous.) Because only five internal peptides were mapped by Edman degradation, it is not known precisely where the region of APV ORF1 that encodes the structural proteins begins. Remote homology searches (HHpred, Phyre 2) map amino acids 1526–2093 of the ORF1 polyprotein to the RdRps of members of the picorna-like virus superfamily (including the families *Caliciviridae* and *Picornaviridae*). Similarly, amino acids 2417–2602 of the ORF1 polyprotein map to picorna-like jelly-roll fold capsid proteins. Thus, if the structural proteins are translated from a sgRNA, then translation on the sgRNA would be expected to begin in the region of codons 2093–2417 of ORF1. Enhanced synonymous site conservation, which might represent a sgRNA promoter element, appears to begin at around codon 2079 of ORF1. The first available AUG codon for structural protein translation initiation downstream of this site is at ORF1 codon 2154 (strong context, G at –3 and G at +4; APV genomic coordinates 6727–6729). Initiation here on a sgRNA would lead to the synthesis of a 53-kDa polypeptide for non-frameshift translation. This is somewhat larger than the 33K virion protein observed by van der Wilk et al. [2], suggesting that the polypeptide may be N-terminally cleaved, perhaps to release a predicted dsRNA binding protein (dsRBP) domain ('*' in Fig. 2A; see also below) [30].

By analogy to *Murine norovirus* and group I sapoviruses, the extended nature of the conserved region between APV and RAAV suggests that it may represent not just a sgRNA promoter, but also one or more overlapping genes. An inspection of the positions of stop codons in the +1 and +2 reading frames relative to ORF1 revealed the presence of two consecutive ORFs (X1, X2) in the +1 reading frame that together cover most of the 3'-extent of the conserved region (Fig. 2A). ORFs X1 and X2 begin, respectively, at the first and third AUG codons on the predicted sgRNA (APV genomic coordinates 6716–6718 and 6968–6970), while the second AUG codon is in the ORF1 frame and is the likely initiation site for structural protein synthesis as mentioned above (APV genomic coordinates 6727–6729). The first AUG codon has a weak context, thus allowing for leaky scanning, at least to the second AUG. These AUG codons are conserved also in RAAV, as is the absence of other intervening AUG codons.

Thus we propose a model for APV in which (i) an sgRNA is produced and (ii) the structural proteins, and potentially two accessory proteins encoded by overlapping ORFs X1 and X2, are translated from the sgRNA (Fig. 2A). Together, these hypotheses would explain the unusual region of conservation in the APV-RAAV alignment. Although these hypotheses were not experimentally tested in this work, the comparative genomic analysis of APV and RAAV is relevant to our experimental analysis of SINV-3 (below), a species for which limited sequence data (just two closely related sequences) currently precludes a similar comparative genomic analysis.

### SINV-3 utilizes ribosomal frameshifting in the expression of its structural proteins

**Figure 3:**
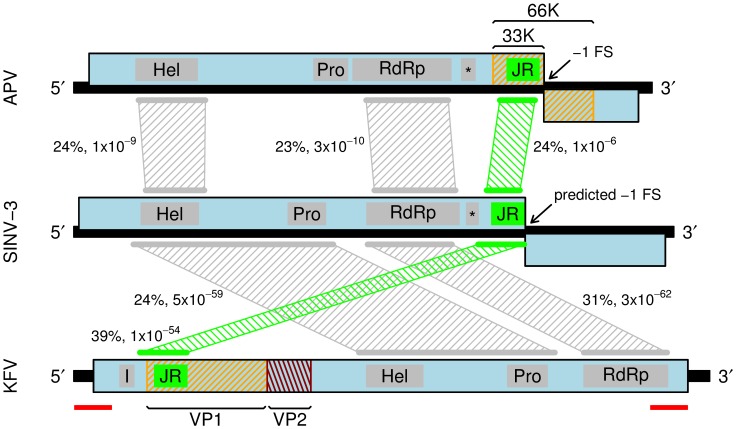
Comparison of APV, SINV-3 and KFV genome organizations. Mappings between homologous regions by simple blastp analysis are indicated with hatched trapezoids. Numbers indicate percentage amino acid identities and blastp e-values (e-values depend on subject database size and other variables so should be taken as indicative rather than absolute). SINV-3 is most similar to KFV despite KFV having a different genome organization. Predicted jelly-roll fold domains (JR) are indicated in bright green. Grey rectangles indicate the extent of domain sequences recognized by HHpred. Predicted dsRNA binding protein domains are indicated with an asterisk. Previously identified APV and KFV structural proteins are indicated with orange and red hatchings (see Fig. 1). Red bars indicate an unusual 638-nt almost-exact repeat sequence in the KFV published genome sequence (DQ112227.1).

As described above, the SINV-3 genome contains two long ORFs, with the product of the 5' ORF containing canonical motifs for the helicase, protease and RdRp domains, but the structural proteins have not been previously mapped. Interestingly, blastp analysis revealed similarity between the C-terminal end of the SINV-3 ORF1-encoded polyprotein (downstream of the RdRp motifs) and the N-terminal one third of the KFV VP1 capsid protein, and also the 33K capsid protein of APV (Fig. 3). Sequence similarity between KFV VP1 and APV structural proteins has been previously noted [5]. Analysis with both HHpred and Phyre 2 further revealed that the C-terminal approximately 194 aa of the SINV-3 ORF1 polyprotein maps to the canonical eight-stranded β-barrel (jelly-roll fold) capsid shell proteins of picornaviruses and caliciviruses ('JR' in Fig. 3). Similar to APV, HHpred also predicted a dsRBP domain ('*' in Fig. 3) just upstream of the jelly-roll domain. The similar genomic location in SINV-3 and APV of the sequence encoding the jelly-roll domain suggested that SINV-3 might, like APV, encode a capsid extension within ORF2 and use ribosomal frameshifting to express the extension as a fusion with the ORF1-encoded capsid protein.

Consistent with this hypothesis we observed that, although the first ORF2-frame AUG codon is some 474 nt 3' of the ORF1 stop codon, the maximal (stop-codon-to-stop-codon) ORF2 extends far upstream and in fact overlaps the 3' end of ORF1 in the -1 reading frame. The overlap between ORF1 and the extended ORF2 comprises just the sequence U_GAU_UUA_AAC_UGA (underscores separate ORF1 codons). This sequence contains a canonical slippery heptanucleotide for -1 frameshifting, namely U_UUA_AAC, the same slippery sequence that is present in APV and RAAV [2], [3], [21].

**Figure 4:**
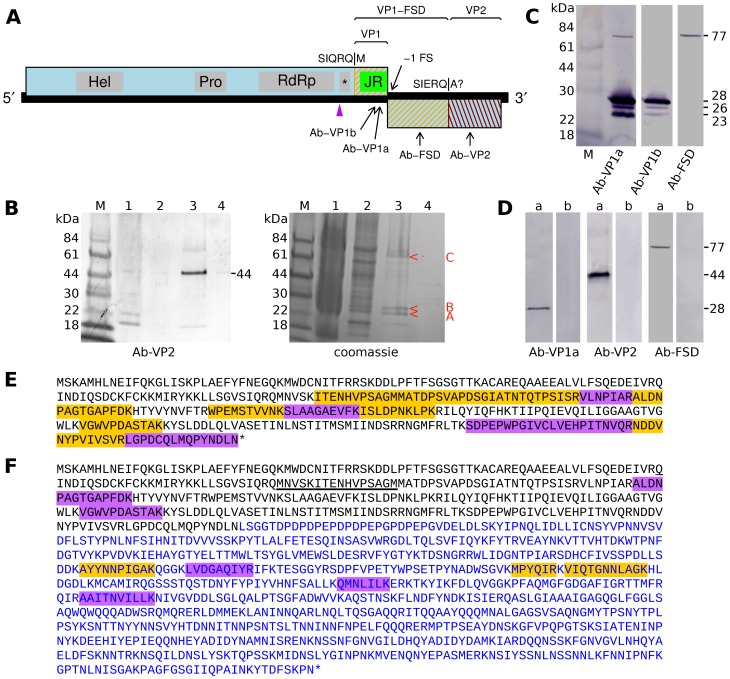
Analysis of SINV-3 virion proteins. **A.** Map of the SINV-3 genome indicating the locations of antibody 14-aa peptide antigens, the locations of structural proteins VP1, VP1-FSD and VP2 as revealed by Western analysis and mass spectrometry, and the N-terminus of VP1 as revealed by mass spectrometry. The cleavage site between VP1-FSD and VP2 has not been definitively localized but is close to the indicated site. **B.** Filtered extracts from SINV-3-infected worker ants were separated by centrifugation in a CsCl gradient (see Methods). Four bands were observed of which band 3 (sedimenting at 1.33 g/ml) was determined by qPCR to contain ∼100-fold more SINV-3 RNA than bands 1, 2 and 4 (Table 1). Analysis of the four fractions by SDS-PAGE (lanes 1–4) and Ab-VP2 Western analysis confirmed the presence of SINV-3 products mainly in fraction 3 (left, lane 3). Coomassie staining (right, lane 3) revealed specific products (i.e. relatively more intense in lane 3 than in other lanes) migrating at 77 kDa and in the 23–28 kDa range (red arrow heads). It is possible that other fractions (lanes 1, 2, 4) contain unrelated viruses present in the sample preparations. **C.** Western analysis of purified virion proteins with Ab-VP1a, Ab-VP1b and Ab-FSD, revealing VP1-related structural proteins. **D.** Western analysis of microsomal fractions prepared from infected worker ants. Samples from SINV-3-infected (a) and uninfected (b) ants were blotted and probed together and intervening lanes removed *in silico* for clarity. **E.** Tryptic peptides identified by mass spectrometry of band B (Fig. 4B, right, lane 3). The sequence region shown corresponds to the the C-terminal end of the ORF1 polyprotein, beginning from the purple arrow head in Fig. 4A. Peptides detected by mass spectrometry are highlighted; multiple highlight colours are used simply to distinguish adjacent peptides. **F.** Tryptic peptides identified by mass spectrometry of band C (Fig. 4B, right, lane 3). Black text represents amino acids encoded by the 3' end of ORF1; blue text represents amino acids encoded by ORF2. The N-terminal-most peptide detected in a chymotryptic digest of a gel slice containing the ∼77 kDa product is underlined; this was also the only N-terminally acetylated peptide detected.

To test the frameshifting hypothesis, we obtained polyclonal Abs to various 14-aa predicted antigenic peptides in the C-terminal end of the ORF1 product and in the ORF2 product (Fig. 4A). SINV-3 virions were purified and the proteins were separated by SDS-PAGE, and subjected to Western analysis and Coomassie staining (Fig. 4B and Fig. 4C). Ab-VP1a, raised against a peptide in the jelly-roll domain encoded within the 3' end of ORF1, recognized four products in purified virions, with masses approximately 77, 28, 26 and 23 kDa (Fig. 4C), with the 28 kDa product giving the strongest signal, and the 77 kDa product giving the weakest signal. Ab-VP1b also recognized 28, 26 and 23 kDa products, but with lower sensitivity, and, consistent with this, the 77 kDa product was not detected with this Ab. The low mass products are consistent in size with termination at the ORF1 stop codon, encompassing the jelly-roll domain (∼21 kDa) and flanking polypeptide sequence. We collectively call these products VP1. The reasons for the mass differences between these related products remain to be investigated and may involve post-translational modifications, cleavage (e.g. during virion maturation), or degradation during sample preparation. Moreover the relative abundances of the different VP1-related products was observed to vary between different sample preparations (cf. Fig. 4B and Fig. 4C). It is worth recalling that three low mass VP1-related products (33K, 24K, 23K) were also observed for APV, again with the most massive being the most abundant [1], [2].

Ab-FSD recognized a product of ∼77 kDa but no products of lower mass. Assuming that Ab-VP1a and Ab-FSD recognize the same 77 kDa product in purified virions, then this product must correspond to a transframe fusion (orange hatching in Fig. 4A) as expected from the ribosomal frameshifting hypothesis. We call this product VP1-FSD, where 'FSD' stands for 'Frame Shift Domain'. The mass of VP1-FSD is too small to encompass VP1 fused to the entire ORF2 product so the ORF2 polypeptide must be cleaved upstream of the Ab-VP2 antigen. Consistent with this, the major product detected by Ab-VP2 had a calculated mass of ∼44 kDa (Fig. 4B, lane 3). We call this product VP2 (red hatching in Fig. 4A). Together, the FSD domain (77 kDa minus 28 kDa) and VP2 (44 kDa) are sufficient to account for the entire ORF2 polypeptide (91 kDa). The identity of a higher mass product in the Ab-VP2 Western blot (very faint band), and a similarly sized product previously observed in Ab-VP2 Western blots of crude ant lysates [40], was not determined but it may represent an aberrant cleavage form.

The VP1-FSD product appears to be much less abundant in virions than VP1 since the band migrating at 77 kDa is much fainter than that migrating at 28 kDa in the Ab-VP1a Western blot (Fig. 4C). However the ratio cannot be accurately estimated from Western analysis because transfer efficiencies may vary for these two very differently sized proteins. Densitometry of a Coomassie-stained SDS-PAGE (Fig. 4B) and normalization by protein size suggested an approximate virion molar ratio of four to five for the non-frameshift (bands A and B) to frameshift (band C) products. Interestingly, a band corresponding to the 44 kDa VP2 product was not obvious, or only very faint, in Coomassie-stained gels suggesting that VP2 may be largely lost during purification, or that only a fraction of expressed VP2 is associated with virions (Fig. 4B, lane 3).

The antibodies exhibited high background and weak sensitivities against crude worker ant homogenates. However, microsomal preparations yielded consistent 28-, 44- and 77-kDa specific detections with Ab-VP1a, Ab-VP2 and Ab-FSD, respectively, presumably due to concentrating viral proteins and eliminating cellular proteins causing the high background (Fig. 4D).

In order to more precisely map the structural proteins, gel slices containing virion proteins were subjected to tryptic digest and liquid chromatography-tandem mass spectrometric (LC/MS/MS) analysis. A gel slice containing the product migrating at 28 kDa (Fig. 4B, band B) contained peptides that mapped to the 3' end of ORF1 (Fig. 4E). On the other hand, a gel slice containing the product migrating at 77 kDa (Fig. 4B, band C) contained both peptides that mapped to the 3' end of ORF1 and peptides that mapped to the 5' half of ORF2 (Fig. 4F). The expected C-terminal peptide of VP1 was detected (Fig. 4E), but the other terminal peptides of VP1 and VP1-FSD were not determined. This could be due to the relevant tryptic peptides being too small or too large, or simply due to limited coverage. In a further attempt to determine cleavage sites, a gel slice containing the product migrating at 77 kDa was subjected to chymotryptic digest followed by LC/MS/MS. In this analysis, the N-terminal-most peptide mapped to the virus genome was MNVSKITENHVPSAGM (underlined, Fig. 4F). This was also the only N-terminally acetylated peptide detected. N-terminal acetylation is a common modification and would explain why our previous attempts to determine the structural protein N-termini by Edman degradation found them to be N-terminally blocked (data not shown). Thus MNVSK… likely represents the N-terminus of VP1-FSD. Translation of VP1-FSD could involve independent initation on the AUG codon corresponding to the N-terminal methionine. However we consider this unlikely due to the larger size of the detected sgRNA (see below) and the weak initation context of this codon (C at -3, A at +4). Alternatively, this represents a proteolytic processing site. The adjacent glutamine amino acid (underlined in SIQRQ|M) is consistent with cleavage by a picorna-like protease. The C-terminal peptide of VP1-FSD remained elusive, but consideration of the measured sizes of VP1-FSD and VP2, suggested SIERQ|A as one possible cleavage site (Fig. 4A).

Together these results suggest a model whereby the main capsid protein (VP1) is encoded by the 3' end of ORF1; an additional, much less abundant, capsid protein (VP1-FSD) is encoded by the 3' end of ORF1 together with the 5' half of ORF2, translated as a transframe fusion via ribosomal frameshifting; and a minor virion-associated protein (VP2) is encoded by the 3' half of ORF2.

### SINV-3 produces a subgenomic RNA during infection

**Figure 5:**
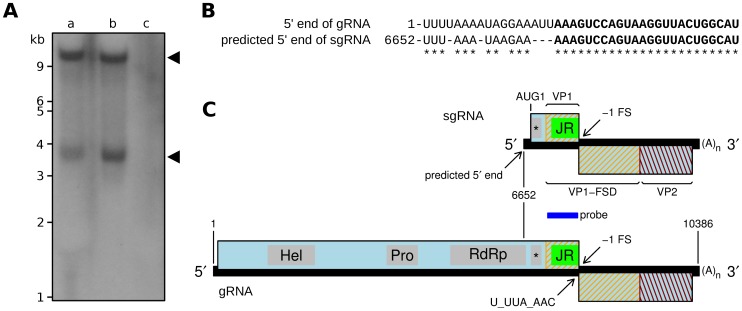
Detection of a sgRNA species in SINV-3-infected ants. **A.** Northern blot of (a) total RNA and (b) mRNA purified from SINV-3-infected *Solenopsis invicta* worker ants, and (c) total RNA purified from uninfected *Solenopsis invicta* worker ants. **B.** Alignment of the SINV-3 genomic and putative subgenomic 5' termini. A 24-nt perfect repeat sequence is indicated in bold. Genomic coordinates are indicated at left. **C.** Map of the SINV-3 genome indicating the sgRNA with its predicted 5' terminus and corresponding translation products. The region covered by the probe used in the Northern analysis is indicated by a dark blue bar.

Given that APV appears likely to employ a sgRNA for bulk structural protein expression, we sought to determine whether SINV-3 might also produce a sgRNA. There are currently only two SINV-3 sequences available (GenBank accessions FJ528584 and GU017972) and, with 96% nucleotide identity, they are too similar for the comparative analyses that we used for APV and RAAV. Instead, Northern analysis was conducted with total RNA and poly(A)-selected RNA purified from SINV-3-infected *Solenopsis invicta* worker ants, and a probe corresponding to SINV-3 genomic coordinates 7164–7805. The Northern analysis revealed two RNA species, one consistent in size with the 10.4-kb genomic RNA, and the second ∼3.8 kb in size (Fig. 5A).

Further sequence analysis revealed a perfect repeat of a 24-nt sequence, AAAGUCCAGUAAGGUUACUGGCAU, at SINV-3 genome coordinates 18-41 (within the 91-nt 5' UTR) and at 6664-6687 (Fig. 5B). The probability for a 24-nt repeat to occur by chance in a 10386-nt genome is ∼1.9×10^−7^. This suggests that the motif is (part of) a functional element that could be involved in the synthesis of genomic and subgenomic RNAs. Furthermore, the 36-nt sequence beginning at nucleotide 6652 and extending to the end of the 24-nt conserved sequence bears considerable similarity to the very 5' end of the genomic RNA (Fig. 5B), suggesting nucleotide 6652 as the correct 5' end of the observed sgRNA, which would then have size 3735 nt plus poly(A) tail. We also attempted to experimentally map the 5' end of the sgRNA with 5' RACE, using RNA treated with proteinase K in order to remove any 5'-linked VPg protein. These experiments yielded four sequences, one of which indicated a start site at nucleotide 6651 (the remaining three sequences were much larger and likely from genomic RNA). Although APV lacks a 24-nt exact repeat sequence, the 35-nt sequence (CGAAA…) starting at genomic nucleotide 6536 bears >70% identity to the 5' terminus of the genome, suggesting 6536 as a possible sgRNA start site in APV. Similarity between genomic and subgenomic 5'-termini is a characteristic also exhibited by caliciviruses [31], [32].

In SINV-3, translation of a sgRNA starting around genomic nucleotide 6652 would begin at the AUG at genomic coordinates 6800–6802 (strong context, A at –3). Translation from this site to the ORF1 stop codon would produce a 38.4 kDa polyprotein (shown in Fig. 4E), which is significantly larger than the 28 kDa VP1 that maps to the 3' end of ORF1. Thus, similar to APV, the sgRNA appears likely to encode a leader protein - corresponding to the predicted dsRBP domain - upstream of the structural proteins ('*' in Fig. 5C), however this remains to be experimentally confirmed. This domain has the same predicted fold as the 1A suppressor of silencing protein of *Drosophila C virus* (family *Dicistroviridae*) [33] and could potentially be involved in suppression of silencing [30], [34]. Members of the calicivirus genus *Vesivirus* also encode a leader protein upstream of the capsid on their sgRNA that is cleaved by the viral protease [35], [36], though these do not appear to be related to the putative SINV-3 leader protein. Neither HHpred nor Phyre 2 detected any predicted structural similarities to known proteins for any part of the polypeptide encoded by ORF2. Thus both SINV-3 and APV appear likely to produce a sgRNA encoding polyproteins dsRBP-VP1 and, via ribosomal frameshifting, dsRBP-VP1-FSD-VP2.

## Discussion

The above observations suggest that SINV-3 and APV may have a capsid protein domain structure similar to caliciviruses, with capsid proteins containing a single picorna-like jelly-roll domain to which is attached an additional domain (or domains) that form protrusions outside of the virion shell. Indeed electron micrographs revealed apparent protrusions on SINV-3 virions [6]. While every calicivirus jelly-roll shell domain is attached to a protruding domain (1∶1 stoichiometry), frameshift expression in SINV-3 and APV means that only a relatively small proportion of jelly-roll domains carry a protruding domain (a similar phenomenon has been noted in cryoelectron microscopy reconstructions of KFV; see below). In SINV-3, a polyproline sequence (PDPDPEPDPDPEPGPDPEP) is encoded just downstream of the frameshift site. Such polyproline sequences may form interdomain linkers and indeed a similar polyproline sequence, (PX)_N_, is encoded in luteoviruses (non-enveloped *T* = 3 plant viruses) just downstream of the stop-codon readthrough site that is responsible for appending an extension domain onto a proportion of their capsid proteins [37], [38], [39]. Caliciviruses and APV lack the polyproline sequence, though caliciviruses have a different hinge peptide between the shell and protruding domains [18].

The SDS-PAGE of purified virion proteins, Western analysis, and mass spectrometry described above indicate that the SINV-3 ORF2-encoded polypeptide is cleaved midway (Fig. 4A). Due to frameshift expression, the N-terminal half is fused to the VP1 jelly-roll domain to produce the VP1-FSD protein. Interestingly, the C-terminal half (VP2) is also virion-associated but was observed to be much less abundant in purified virions than VP1-FSD (Fig. 4B). It is possible that both N- and C-terminal domains are present in the same ratio in native virions but VP2 is lost during purification. It is also possible that the N- and C-terminal domains are not normally cleaved as this could simply be an artifact of purification; however this appears unlikely as the antibody to this region (Fig. 4A) also detects the 44-kDa product in crude ant lysates [40]. Previous data demonstrate that the APV ORF2-encoded polypeptide is also cleaved, as the size of the transframe fusion protein (∼66 kDa) is too small to encompass the entire sequence (Fig. 1). However it is not known whether or not the APV C-terminal domain is associated with APV virions.

As discussed above, KFV virions contain two major products of 75 kDa and 29 kDa in the approximate ratio 1∶5, the capsid shell appears to comprise 60 copies of an asymmetric unit of ∼75 kDa, and the capsid bears protuding domains of ∼70 kDa (masses based on volumetric comparisons with other virus structures). It was proposed that VP1 might represent the protruding domain, with a footprint in the shell, while the bulk of the shell might comprise VP2 [5]. Homology between KFV VP1 and the APV 33K/66K proteins, particularly in 33K has been noted previously, and there is even higher similarity with SINV-3 (Fig. 3). Although the jelly-roll fold domain had not been previously identified in KFV, application of HHpred to KFV confirmed that the N-terminal one third of VP1 harbours a predicted picorna-like jelly-roll domain (Fig. 3). On the other hand, KFV VP2 showed no homology by HHpred, Phyre 2 or blastp to any other virus protein. Thus APV, SINV-3 and KFV all have two major virion proteins, with the smaller protein (∼25 kDa) being approximately four to ten times as abundant as the larger protein (∼75 kDa). SINV-3 and KFV have similar particle morphologies (icosahedral with protrusions; similar size and density). In SINV-3 and APV, the smaller protein largely comprises a jelly-roll domain, while the larger protein contains the smaller protein as its N-terminus and contains a large C-terminal extension domain. These observations lead us to hypothesise that (i) the KFV shell actually comprises 180 copies of the N-terminal third of VP1 (not VP2), which, by alignment to SINV-3 has a mass of ∼25 kDa, consistent with the cryoelectron microscopy reconstruction; (ii) the KFV turret domain corresponds to the C-terminal two thirds of VP1 and is homologous to the frameshift domains of SINV-3 VP1-FSD and APV 66K; and (iii) like SINV-3 VP2, and presumably also the protein encoded by the 3' half of APV ORF2, KFV VP2 is a third virion-associated protein.

It is interesting that SINV-3 has significantly higher amino acid identity to KFV than to APV in the non-structural polyprotein and the jelly-roll capsid domain, yet its genome organization and the number and expression level of the structural proteins is APV-like (Fig. 3; Fig. 4C). In particular, SINV-3 expresses low amounts of VP1-FSD relative to VP1 via frameshifting, yet in KFV only the equivalent of full-length VP1-FSD is expected to be produced (perhaps the VP1 shell domain on its own might then be derived by cleavage, though potentially an undetected and highly efficient ribosomal frameshift to divert a high proportion of ribosomes into a termination codon in an alternative reading frame could achieve the same effect). A number of Transcriptome Shotgun Assembly (TSA) projects have been performed on insect subjects, and frequently RNA viral sequences are assembled alongside host mRNA sequences. Blast (tblastn) querying of APV, SINV-3 and KFV amino acid sequences against the NCBI TSA database revealed a number of related sequences. Most are fairly short but GAJX01000318.1 (10269 nt; ∼25–30% aa identity to APV in alignable regions; isolated from *Clavigralla tomentosicollis*, an hemipteran insect) and GAPE01025462.1 (5536 nt; ∼32% aa identity to SINV-3; isolated from *Meligethes aeneus*, a beetle) potentially represent considerable proportions of viral genomes. GAJX01000318.1 is based on Roche 454 sequencing, which is prone to frameshift sequencing errors, and the presumed polyprotein ORF1 is split into a three major ORFs and a couple of minor ORFs. GAPE01025462.1 is based on Illumina HiSeq sequencing and contains a single unbroken ORF covering the entire sequence. Interestingly both GAJX01000318.1 and GAPE01025462.1 span the equivalent of the APV or SINV-3 ORF1-ORF2 junction, yet lack a change in reading frame at this location. In other words, like KFV, translation of GAJX01000318.1 or GAPE01025462.1 would fuse an extension domain to every copy of the jelly-roll domain. It should be noted that, over the course of evolution, sequences derived from viruses (including RNA viruses) can become integrated into host genomes where they may be exapted [41]. Such sequences may be transcribed and potentially could play protective roles (e.g. endogenous expression of a defective virus structural protein could interfere with virion assembly under infection with a related virus). Thus it is not certain that GAJX01000318.1 or GAPE01025462 derive from, or are representative of, *bona fide* viruses. If they are then, together with KFV, they provide evidence for plasticity in the stoichiometry of the capsid to capsid-extension domains in this virus clade. Given the huge divergences between APV, SINV-3, KFV and these two TSA sequences (typical amino acid identities 24–39% in alignable regions) such plasticity is perhaps not too surprising.

On the other hand, both GAJX01000318.1 and GAPE01025462.1 share genome synteny with APV and SINV-3, in particular, with the jelly-roll domain being encoded downstream of the RdRp domain. The different organization in KFV (structural proteins encoded upstream of the RdRp domain) is so far unique within the clade. Curiously, nucleotides 17–654 of the published KFV sequence (DQ112227.1) are an almost exact duplicate of nucleotides 10007–10644 (only 3 mismatches in 638 nucleotides) (red bars in Fig. 3). This duplication coincides with the site of genome rearrangement relative to SINV-3 and suggests to us that the KFV genome may actually be organized similarly to those of APV and SINV-3, with the current KFV sequence possibly needing to be split at a point between the regions encoding VP2 and the Hel domain.

Single-stranded positive-sense RNA viruses in the families *Picornaviridae*, *Secoviridae*, *Iflaviridae*, *Dicistroviridae* and *Marnaviridae* have been formally assigned to the order Picornavirales [42], [43]. Where investigated, members of the order have (i) a non-segmented or occasionally bipartite genome; (ii) an absence of sgRNAs; (iii) a polyprotein expression strategy with most cleavage events mediated by one or more virus-encoded proteases; (iv) a superfamily III helicase (or NTPase), a chymotrypsin-like protease, and a superfamily I RdRp encoded sequentially in this order; and (v) non-enveloped icosahedral virions with pseudo *T* = 3 symmetry (60 units each containing three distantly related ∼25 kDa jelly-roll domains). Although it had been proposed previously that members of the family *Caliciviridae* also be included in a Picornavirales order [44], a number of characteristics set them aside, including (i) the presense of a sgRNA for structural protein expression; and (ii) virions have true *T* = 3 symmetry (180 copies of a single jelly-roll domain).

In an in-depth phylogenetic analysis of the RdRp and helicase domains of picorna-like viruses, Koonin et al. found that the APV and KFV RdRps clustered more closely with picornavirus RdRps than with calicivirus RdRps, but the APV and KFV helicases clustered more closely with calicivirus helicases than with picornavirus helicases [45]. On the other hand Hartley et al. found that APV and KFV RdRps clustered loosely with caliciviruses over picornaviruses, though the tree topology is difficult to resolve at this depth [5]. With regards to the structural proteins, the following features of APV and SINV-3 appear more calicivirus-like: (i) genomes encode a single picorna-like jelly-roll domain instead of three; (ii) structural proteins are encoded at the 3' end of the genome; (iii) SINV-3 and likely also APV produce a sgRNA from which the structural proteins may be expressed, even though the structural proteins can also be translated from the genomic RNA; and (iv) besides the jelly-roll shell domain, the structural proteins also include additional domains that appear to form protrusions. Whether the protein encoded by the 3' half of ORF2 in APV and SINV-3 is in any way related (functionally or evolutionarily) to the calicivirus VP2 protein remains to be seen. Van der Wilk et al. noted that the C-terminal region of the APV ORF2 product had low similarity to the calicivirus ORF3 product [2]; and we also have observed local similarities between the VP2 proteins of APV, SINV-3 and caliciviruses, though these similarities are not obviously statistically significant. The presence and/or size of a VPg in APV, SINV-3 or KFV has not been determined. Unlike most caliciviruses, APV, RAAV and SINV-3 have relatively long 5' UTRs (267 nt, 275 nt and 91 nt). It is not known whether or not these UTRs harbour IRES elements. However, the SINV-3 5' UTR contains no internal AUGs and is GC-poor (37% GC), suggestive of 5'-end dependent rather than IRES-dependent translation initiation. Similarly, the APV and RAAV 5' UTRs each contain just a single internal AUG codon (very weak context, 8–10 codon ORF), and have 30% GC content, so are not expected to be greatly inhibitory to 5'-end dependent scanning, although their long length suggests additional functionality. The status of the KFV 5' UTR is uncertain as the published 5' UTR sequence (DQ112227.1) largely comprises a direct repeat of nucleotides 10010-10644, including part of the RdRp-encoding sequence (see above).

We have presented new experimental and bioinformatic data that tie together the gene expression strategies of SINV-3 and APV and suggest that KFV may be more similar to these viruses than previously appreciated. Nonetheless these viruses are highly divergent from each other and it will be necessary to await the acquisition of new sequences for more species in the clade in order to better define the phylogenetic relationships of these viruses to each other and to other viruses in the picorna-like virus superfamily. Blast analysis using the NCBI TSA database indicates that SINV-3- and APV-like sequences are widespread in insect hosts. It would also be useful to investigate the presence and size of VPg proteins in this clade, acquire structural information for SINV-3 and/or APV virions, and determine the role of the VP2 protein.

## Materials and Methods

### Ethics statement

No specific permits were required to collect field specimens because they did not occur in locations protected in any way. The field collections did not involve endangered or protected species.

### Computational analyses

Sequences were processed using EMBOSS [46] and analyzed using blast [47], T_Coffee [48], HHpred [49] and Phyre 2 [50]. Genomic coordinates are quoted with respect to AF024514.1 (APV), DQ286292.1 (RAAV), DQ112227.1 (KFV) and FJ528584.1 (SINV-3). For the synonymous site conservation analysis, sequences were aligned using ClustalW [51] and analyzed as described previously [22].

### Purification of SINV-3 virions

As there is currently no cell culture system for SINV-3, 50 g of ant workers from a SINV-3-infected fire ant colony (testing negative for SINV-1 and SINV-2) were homogenized for 2 min in a Waring blender (high setting) in 200 ml of NTB buffer (10 mM Tris, pH 7.25, 0.1 mM NaCl). Homogenate was filtered through cheesecloth, then extracted with 2x volume chloroform for 30 min with gentle shaking, and centrifuged for 10 min to separate the phases. The supernatant was removed and applied to a 1.2/1.5 g/ml CsCl step gradient and centrifuged at 195,000 *g* for 2 hr. The interface was recovered by pipette, equilibrated to 1.35 g/ml CsCl and centrifuged for 17 hr at 330,000 *g*. Three bands were observed: upper (1.217 g/ml), middle (1.266 g/ml), and lower (1.296 g/ml). Each was removed by pipette, brought to 1.35 g/ml CsCl, and centrifuged again at 330,000 *g* for 17 hr. Each yielded a single band again with the exception of the lower band which yielded two (lower and lower 2). Each of these bands was recovered by pipette, diluted in NTB and centrifuged at 195,000 *g* for 2 hr. The pellets were resuspended in 300 μl of NTB. RNA was extracted from each fraction with Trizol and qPCR conducted. The results of each band are summarized in Table 1.

**Table 1:** Analysis of fractions obtained during virion purification.

Fraction (band)	Density (CsCl)	SINV-3 (GE)*
Upper (1)	1.293	4.48×10^4^
Middle (2)	1.274	2.87×10^4^
Lower (3)	1.330	2.37×10^6^
Lower 2 (4)	1.360	4.20×10^4^

* SINV-3 genome equivalents per ng total RNA by qPCR.

### Preparation of microsomes

Worker ants (∼0.25 g) from SINV-3-infected colonies were homogenized in 10 ml of 0.1 M sodium phosphate buffer, pH 7.6, containing 1 mM EDTA and 1 mM phenylmethylsulfonyl fluoride using a motor-driven Potter-Elvehjem Teflon pestle and glass mortar. The homogenate was filtered through cheesecloth, then centrifuged at 1000 *g* for 15 min. The supernatant was centrifuged at 105,000 *g* for 1 hr. The resulting microsomal pellet was suspended in 1–3 ml of sodium phosphate buffer, 0.1 M, pH 7.6.

### Antibodies

Polyclonal rabbit antibodies were prepared by GenScript for the following peptide sequences: DSRRNGMFRLTKSD (Ab-VP1a); VPDASTAKKYSLDD (Ab-VP1b); IGAKQGGKLVDGAQ (Ab-FSD); and ERMPTPSEAYDNSK (Ab-VP2; previously described in ref. [40]). A fifth polyclonal Ab raised against ALDNPAGTGAPFDK (Ab-VP1c) proved ineffective.

### Analysis of virion proteins and Western blotting

Homogenates or tissue preparations were denatured (Laemmli method SDS/BME/heat 5 min) and applied in duplicate to 4-20% gradient SDS-PAGE gels (TGX, Biorad). After electrophoresis, one gel was stained with Coomassie (GelCode) and the other electroblotted onto polyvinylidene fluoride (PVDF) membrane and probed with each antibody at the following optimized concentrations: Ab-VP1a (38 μg/ml), Ab-VP1b (4 μg/ml), Ab-VP1c (70 μg/ml), Ab-FSD (103 μg/ml), Ab-VP2 (3 μg/ml). The PVDF membrane was blocked in a TBS (tris buffered saline; 20 mM Tris-HCl, 500 mM NaCl, pH 7.5) +1% BSA solution overnight at 4°C. Blots were exposed to primary antibody preparations for 2 hr at room temperature (40 rpm) and then washed. The blots were then probed with secondary antibody (10,000-fold dilution), goat anti-rabbit conjugated with alkaline phosphatase for 1 hr. Membranes were washed for several minutes with BCIP (5-bromo-4-chloro-3-indolyl-phosphate) and NBT (nitro blue tetrazolium) for the colorimetric detection of alkaline phosphatase activity. Once bands were detected on the blot, the reactions were terminated by rinsing with deionized water. For Coomassie-stained gels, band intensities were estimated by densitometry using TotalLab TL100 software (Nonlinear Dynamics, Newcastle upon Tyne, UK). Pixel volumes were used to estimate the intensities of each band.

### Mass spectrometry

Coomassie-stained products were excised from the gel, subjected to in-gel trypsin or chymotrypsin digestion, and analyzed by LC/MS/MS. Peptides were identified using the MASCOT search engine using a custom database including the entire SINV-3 ORF1 and ORF1-ORF2 polyproteins. Peptide assignments were required to have MASCOT scores greater than 20 and 'expect' values less than 0.0001.

### Northern analysis

Total RNA and mRNA samples were purified from SINV-3-infected and uninfected *Solenopsis invicta* colonies. Total RNA was prepared from 600 SINV-3-infected worker ants using Trizol and the PureLink micro-to-midi total RNA purification system (Ambion). Messenger RNA was subsequently purified from total RNA (100 to 150 μg) with the MicroPoly(A)Purist kit (Ambion) according to the manufacturer’s instructions. The integrity of all RNA preparations was assessed by microfluidic analysis on an Agilent 2100 Bioanalyzer (Agilent, Cary, NC) using the RNA 6000 Nano kit according to the manufacturer’s directions. Northern analysis was completed by Lofstrand Labs Limited (Gaithersburg, MD). RNA samples (16–20 μg total RNA; 1.5–1.8 μg mRNA) and Century Plus and Millenium RNA standards (3 μg; Ambion) were loaded onto a NorthernMax-Gly 1% agarose gel (Ambion) and electrophoresed at 175 V for approximately 2 hr. The gel was equilibrated in 20X SSC for 2 min and then transferred to a Nytran Superchare membrane (Whatman) using a TurboBlotter with 20X SSC overnight. The membrane was UV crosslinked and air-dried. The membrane was pre-hybridized in 6X SSC, 5X Denhardt’s, 0.5% SDS solution at 68°C for 7 hr. The 642-nt probe (corresponding to FJ528584 nt 7164–7805) was synthesized by PCR using oligonucleotide primers p705 (5'-CTGCTGGTATGATGGCAACAGATCCTTCTGT) and p709 (5'-GCTGGCAATCAGGACCAAGTCTAACACTAACAATA), 32P-labeled dCTP and cDNA prepared from SINV-3-infected colonies with oligonucleotide primer p709 as template. The specific activity of the gel-purified probe was 1×109 dpm/µg. The membrane was hybridized at 68°C for 18 hr. The membrane was washed in 2X SSC + 0.1% SDS at 68°C 3 times every 20 min. The membrane was autoradiographed for 20 hr and then developed.

### 5' RACE

5' rapid amplification of cDNA ends was completed by anchored PCR [52]. Total RNA was purified from worker ants with active SINV-3 infections using the Trizol method. RNA (100–500 μg) was digested with proteinase K (40 μg) at 37°C for 30 min. The digested RNA was purified by precipitation with sodium acetate/isopropanol to eliminate proteinase K. Complementary DNA was synthesized from the RNA templates using an oligonucleotide primer to genome nucleotides 6911–6945. Complementary DNA was polycytidylated with terminal deoxynucleotidyl transferase (Invitrogen) in the presence of 2 mM dCTP, and PCR was completed with an abridged anchor primer (Invitrogen) and a SINV-3-specific primer (genome nucleotides 6816–6847). Gel-purified amplicons were ligated into pCR4-TOPO vector, transformed into TOP10 competent cells (Invitrogen) and sequenced by the Interdisciplinary Center for Biotechnology Research (University of Florida) by the Sanger method.
